# Herbal Bioactives Targeting Rho GTPases: A Multi-Targeted Strategy for Mitigating Neuroinflammation in Alzheimer’s and Parkinson’s Diseases

**DOI:** 10.3390/cimb48070694

**Published:** 2026-07-08

**Authors:** Tzong-Shi Wang, I-Shiang Tzeng, Yi-Chyan Chen, Mao-Liang Chen

**Affiliations:** 1 Department of Psychiatry, Taipei Tzu Chi Hospital, Buddhist Tzu Chi Medical Foundation, New Taipei City 231016, Taiwan; tswang1014@gmail.com (T.-S.W.); yichyanc@gmail.com (Y.-C.C.); 2 Department of Medical Research, Taipei Tzu Chi Hospital, Buddhist Tzu Chi Medical Foundation, New Taipei City 231016, Taiwan; xdd05082@tzuchi.com.tw

**Keywords:** Rho signaling, neuroinflammation, bioactives, Alzheimer’s disease, Parkinson’s disease

## Abstract

Neuroinflammation plays an essential role in the pathogenesis of several associated brain diseases, including neurodegenerative disorders (Alzheimer’s disease (AD), Parkinson’s disease (PD), multiple sclerosis (MS)), and traumatic brain injury (TBI). In these diseases, persistent microglial and astrocyte aggregates, elevated proinflammatory cytokines, and oxidative stress drive neuronal injury and cognitive disability. Rho GTPases, in particular the Rho family members Ras homolog family member A (RhoA), Ras-related C3 botulinum toxin substrate 1 (Rac1), and cell division control protein 42 homolog (CDC42), regulate neuroinflammation, cytoskeletal dynamics, immune responses, and the maintenance of BBB integrity. These proteins are involved in many neuropathological diseases due to dysregulation, making them interesting therapeutic targets. Bioactives used in herbal care have attracted interest for their ability to influence neuroinflammation and even their anti-neurodegenerative activity. Studies show that flavonoids, alkaloids, polyphenols, and other botanical compounds alter Rho GTPase activity, which, in turn, leads to decreased inflammation. This review critically summarizes current evidence regarding phytochemical regulation of Rho GTPase signaling in neurodegenerative disorders such as Alzheimer’s disease (AD) and Parkinson’s disease (PD), with particular emphasis on the underlying molecular mechanisms, context-dependent signaling responses, and current translational challenges. Furthermore, existing knowledge gaps and future research priorities are discussed to facilitate the development of mechanism-based therapeutic strategies targeting Rho GTPases.

## 1. Introduction

Neuroinflammation is a classical pathological feature of neurological disease, triggering microglial activation, astrocyte pathology, and the hyperproduction of proinflammatory cytokines as promoted by the disease. It is the initial phase of the neurotherapeutic pathway, focusing on targeting glial-mediated neurotoxicity and inflammation mechanisms to alter the immune response toward anti-inflammatory signals. Rho family GTPases—particularly RhoA, Rac1, and CDC42—critically mediate cell signaling pathways that regulate immune responses, oxidative stress, blood–brain barrier (BBB) integrity, and neuronal survival. In various neurodegenerative diseases, such as AD [1,2,3,4], PD [5,6,7], and MS [8,9], these proteins lose their normal regulation, while chronic inflammation drives progressive neuronal destruction. Rho GTPases regulate the dynamics of the actin cytoskeleton [10,11], modulate synaptic plasticity [12,13,14], facilitate neural repair [14,15,16], and promote apoptosis [17,18]. Consequently, they may play a crucial role in neuroinflammatory spectrum disorders.

Various bioactive compounds in herbal medicines effectively regulate Rho-dependent signaling pathways at the BBB. By preventing neuroinflammation induced by the immune destruction of Rho GTPase receptors, these compounds protect the BBB and promote neuroprotection. The compounds flavonoids, alkaloids, and polyphenols, which have anti-inflammatory, antioxidant, and neurotrophic activities, comprise a group of phytochemicals that are potential therapeutic targets for the treatment of brain diseases [19,20,21,22,23].

Although numerous reviews have summarized the neuroprotective effects of bioactives/phytochemicals, few have specifically focused on Rho GTPases as central signaling hubs linking neuroinflammation, cytoskeletal remodeling, oxidative stress, and neuronal survival. Moreover, accumulating evidence indicates that phytochemicals may regulate multiple upstream regulators and downstream effectors of Rho GTPases in a context-dependent manner. Therefore, this review aims not only to summarize existing evidence but also to critically evaluate mechanistic insights, translational limitations, and future directions for Rho-targeted phytochemical therapies.

## 2. Burden of Neuroinflammatory Disorders

The aging population and rapidly increasing life expectancy have led to a global rise in health problems, including neurodegenerative diseases. These diseases, which include AD, PD, and MS, are characterized by progressive neuronal loss and cognitive decline and frequently result in severe disability [24]. Chronic neuroinflammation plays a vital role in neurodegeneration by inducing neuronal dysfunction, oxidative stress, and apoptotic cell death through persistent activation of microglia and astrocytes [25,26,27,28]. Additionally, disruption of the blood–brain barrier accelerates disease progression by enabling peripheral immune cells to infiltrate neural tissue [29]. Existing therapies are usually symptomatic and do not prevent or arrest disease progression; therefore, there is an increasing urgency for new therapeutic approaches. One recently established alternative for a neuroimmunity response is herbal medicine, which modulates neuroinflammatory pathways and offers neuroprotective benefits. Numerous studies have reported bioactive compounds from medicinal plants with anti-inflammatory, antioxidant, and neurotrophic activities. On this basis, they seem to be attractive candidates for buffering neuroinflammation and preserving neuronal integrity.

### 2.1. Neuroinflammation in AD and PD

Multiple pathways, including microglial activation, astrocyte regulation, cytokine release, and oxidative stress, mediate neuroinflammation. It is a common feature contributing significantly to the genesis and development of several diseases, including AD and PD.

#### 2.1.1. Alzheimer’s Disease

In AD, persistent inflammatory responses in neurons increase amyloid-beta (Aβ) levels, tau hyperphosphorylation, and synaptic impairment, ultimately leading to gradual cognitive impairment [24,30]. Activation, migration, and adhesion of neutrophils to the vascular endothelium [31,32] were also facilitated and associated with multiple cellular factors, including C5a, leukotriene B4, platelet-activating factor, and N-formylmethionyl-leucyl-phenylalanine [33]. Neutrophils also aggregate in cerebral blood vessels, particularly around Aβ deposits [34], thereby affecting the BBB and triggering vascular inflammation [35]. Studies demonstrate that neutrophil attachment to blood vessels disrupts the BBB [35] and triggers the production of ROS, inflammatory molecules, tissue proteases, and arachidonic acid derivatives, leading to vascular wall degradation [36]. In addition, αMβ2 integrin Mac-1 demonstrates endothelial cell binding and NET release elicitation [37]. From these pieces of evidence, it emerges that neuroinflammation is a major contributor to the onset of AD.

#### 2.1.2. Parkinson’s Disease

PD is a neurodegenerative disease primarily characterized by the deposition of misfolded α-synuclein within Lewy bodies [38,39], which eventually leads to the degeneration of dopaminergic neurons in the substantia nigra [40]. Microglia, the primary immune cells residing in the central nervous system (CNS), play a crucial role in the neuroinflammatory pathways of Parkinson’s disease (PD). These cells can express various activation states, which fall roughly into two main types: proinflammatory M1 and anti-inflammatory, cytoprotective M2. Microglia can switch between these phenotypes and can even modulate disease development and pathophysiology in response to pathological stimuli, such as aggregated α-synuclein [41]. Meta-molecular data from PD brains have shown widespread microglial activity after death, with activation, particularly in the substantia nigra and other affected areas. Moreover, the amoeboid morphology and the major histocompatibility complex class II (MHC II) molecules that these stimulated microglia express are higher than those of their counterparts, suggesting they are immune-sensitive. Importantly, the degree of MHC II expression correlates with α-synuclein pathogenesis but is not strictly predictive of disease duration or motor symptom severity [5,42]. Several investigations in animal models of PD have confirmed the microgliosis observed in patients [43]. Microglia not only respond to neuronal death but have also been shown in many studies to alleviate α-synuclein cytotoxicity in neurons early in the excitatory phase yet remain reactive to the toxin [44,45]. Microgliosis was reported in general and in human and animal PD models. These models suggest that microgliosis is not a response to neuronal death but rather to early α-synuclein toxicity. Such early activation reflects the role of microglia in pro-degenerative neurodegenerative processes [5]. Proinflammatory cytokines, tumor necrosis factor-alpha (TNF-α) and interleukin-6 (IL-6), modulate dopaminergic neurons, leading to degeneration of the substantia nigra, deterioration of motor symptoms, and disease exacerbation [46,47,48]. Neuroinflammation in PD is a neurodegenerative process initiated primarily by microglial activation in response to misfolded α-synuclein. Cytokines such as TNF-α and IL-6, as well as dopaminergic neuron loss, can also influence the course of disease through a variety of pro- and anti-inflammatory states. Parallel to PD and AD, chronic neuroinflammation drives diseases such as MS, TBI, Huntington’s disease, amyotrophic lateral sclerosis, and stroke, triggering persistent activation of inflammatory pathways and irreparable neuronal damage. Understanding the mechanisms by which neuroinflammation triggers inflammation in these diseases is an important step toward developing targeted therapeutics.

### 2.2. Mechanisms of Neuroinflammation

The immune cells, cytokines, and intracellular signaling pathways associated with neuroinflammation are intricately interconnected. Microglial and astrocytic activation results in the release of proinflammatory cytokines, including TNF-α, IL-1β, and IL-6, that potentiate the inflammatory response and mediate neuronal injury [49,50]. These molecules activate intracellular pathways such as nuclear factor kappa B (NF-κB) and mitogen-activated protein kinases (MAPKs), leading to enhanced oxidative stress, mitochondrial dysfunction, and excitotoxicity, driven by intracellular signaling [50,51].

In addition to cytokine release, neuroinflammation arises from immune cell infiltration, changes in the blood–brain barrier, and glial cell activation. These processes progressively accelerate, inducing sustained neuroinflammation and neurodegeneration. Emerging evidence suggests that interfering with or managing these pathways might uncover potential therapeutic targets to halt or delay neurodegenerative diseases.

### 2.3. The Role of Rho GTPases in Neuroinflammation

The Rho family of GTPases, including RhoA, Rac1, and CDC42, is an important regulator of the cellular signaling pathways in neuroinflammation responses. Such proteins are important for cytoskeletal remodeling, maintaining BBB integrity, regulating immune cell migration, providing neuroprotection, and promoting neuron survival. In several neurodegenerative disorders, the impairment of Rho GTPases and related inflammatory Rho GTPases leads to increased inflammation and neuronal destruction.

#### 2.3.1. RhoA

TNF-α leads to a breakdown of the blood–brain barrier via Rho-kinase- and/or neurokinin 1 receptor-mediated cell dysfunction [52]. The barrier-protective effects of both Y-27632 and CP96345 were attributed to inhibition of RhoA activation and actin stress fiber formation, thereby restoring Zonula occludens-1 plasma membrane localization. Thus, both Y-27632 and CP96345 inhibited RhoA [52]. Methamphetamine is also believed to be capable of activating the RhoA/Rho-associated, coiled-coil-containing protein kinase (ROCK) signaling pathway and inducing BBB disruption [53]. Early studies identified RhoA/ROCK as an inhibitor of axon expansion in the CNS [54], whereas inactivating the Rho signaling pathway promotes CNS axon regeneration [55]. RhoA upregulates neuroinflammation via NF-κB and c-Jun N-terminal kinases (JNK), leading to elevated proinflammatory cytokine release and oxidative stress [56,57]. Increased RhoA/ROCK activity is implicated in AD and PD [58,59,60,61,62], contributing to disease pathology through chronic neuroinflammation and synaptic dysfunction. In brief, the RhoA/ROCK signaling axis is central to the vascularization of AD and PD by promoting disruption of the blood–brain barrier, inhibiting axon regeneration, and inducing neuroinflammation.

#### 2.3.2. Rac1

Rac1 also modulates neuroinflammation. Specifically, Rac1 activity maintains basal autocrine production and signaling of interleukin-6 [63]. Ras and Rho GTPases target the Janus kinase (JAK)/signal transducer and activator of transcription 3 (STAT3) pathway, whereas IL-6 delays STAT3 phosphorylation and activation [64]. A second cause of Rac1-related neuronal damage and oxidative stress [65,66,67] is the activation of nicotinamide adenine dinucleotide phosphate (NADPH) oxidase, which produces reactive oxygen species (ROS). Rac1 potentiates microglial migration and phagocytosis, which, in turn, regulate immune surveillance and the disposal of toxic protein aggregates (such as Aβ) [68,69,70]. Rac1 dysregulation reduces synaptic plasticity and drives cognitive impairment in neurodegenerative diseases [71,72,73]. Taken together, these findings highlight the multiple roles Rac1 plays in regulating neuroinflammation, oxidative stress, and the CNS immune response. Its dysfunction contributes to the molecular pathology of neurodegenerative diseases through synaptic dysregulation, elevated oxidative stress, and abnormal cytokine signaling. Crucially, while overactivation drives oxidative damage, total non-selective inhibition of Rac1 poses a severe risk of impairing essential microglial phagocytosis and aggregate clearance.

#### 2.3.3. CDC42

Studies show that CDC42 activation mediates IL-6-induced inflammation. Blocking CDC42 prevents these cells from responding to IL-6’s pro-invasive effects [74]. The CDC42/ROCK1 pathway also regulates microglial morphology and function, thereby influencing brain immune responses [75]. Also, RhoA, Rac1, and CDC42 were implicated in driving microglial migration to the site of injury at the BBB and in modulating proinflammatory/breakdown-response processes [76]. RhoA, Rac1, and CDC42 proteins encoded by the Rho family affect actin polymerization (both inhibitory and stimulatory), synaptic plasticity, and neuronal cell communication [77]. Endocytosis and intracellular transport are also affected by this, affecting the clearance of neurotoxic aggregates [78,79,80]. These points indicate that perturbations in CDC42 signaling drive synaptic loss and neurodevelopmental disorders. Thus, CDC42 signaling exhibits strict context dependency; while it can facilitate certain pro-inflammatory cascades, its basal activity is indispensable for dendritic spine structural remodeling, meaning that non-selective therapeutic targeting could inadvertently accelerate synaptic loss.

**Figure 1:**
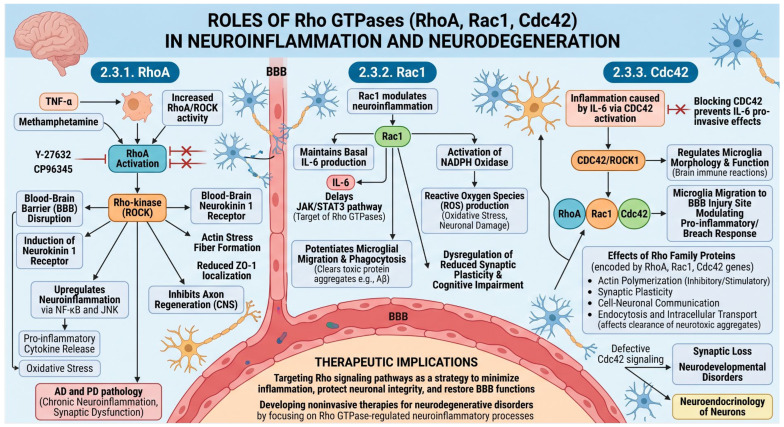
Mechanistic overview of Rho GTPases (RhoA, Rac1, and CDC42) in neuroinflammation and neurodegeneration. The schematic illustrates the signaling pathways regulated by the Rho family of GTPases in the CNS and their contributions to BBB disruption, neuroinflammation, and neuronal decline.

Target Rho as a therapeutic strategy in neuroinflammatory diseases, modulating pathways to minimize inflammation, protect neuronal integrity, and restore BBB function (Figure 1). Focusing on the specific Rho GTPase-regulated pathways that drive neuroinflammatory processes will lead to the development of noninvasive therapies for neurodegenerative disorders.

## 3. Neuroprotective Role of Herbal Bioactives

Nuanced neuroprotective properties have been investigated in medicinal plants, and they are particularly relevant for neuroinflammation and neurodegenerative disease. Numerous bioactive substances present in medicinal plants have anti-inflammatory, antioxidant, and neurotrophic effects that may have therapeutic usefulness in other diseases, including AD, PD, MS, and TBI.

### 3.1. Mechanisms of Action

Herbal compounds exert this targeting via multiple mechanisms across neuroinflammatory pathways.

#### 3.1.1. Modulation of Rho GTPases

Numerous bioactive compounds in plants enhance Rho GTPase activity, with corresponding effects on cytoskeleton dynamics, synaptic plasticity, and blood–brain barrier integrity. Thus, biochemical compounds can activate or suppress the activity of RhoA, Rac1, and CDC42 through either direct structural interference or indirect upstream regulatory networks, thereby causing distinct effects on neuroinflammation and neuronal survival. For example, curcumin has inhibited RhoA/ROCK signaling [81,82] and, in turn, promoted axonal regeneration [83] and reduced inflammation [84,85] while resveratrol acts on Rac1 activity to counteract oxidative stress [86,87]. In addition, flavonoids such as quercetin and epigallocatechin gallate (EGCG) modulate microglial activity and cytokine production by interacting with Rho GTPases [88,89,90]. These results emphasize the therapeutic potential of herbal medicine for inhibiting Rho GTPases in neurological disorders.

#### 3.1.2. Direct vs. Indirect Upstream Modulation of Rho GTPases

A critical mechanistic distinction must be made between phytocompounds that directly interact with Rho GTPases and those that modulate them indirectly via upstream cascades. Accumulating evidence indicates that most herbal bioactives act through indirect upstream modulation. For instance, resveratrol suppresses oxidative stress by activating AMP-activated protein kinase (AMPK), an upstream negative regulator, thereby preventing Rac1 membrane translocation and subsequent NADPH oxidase (NOX) assembly [86]. Similarly, quercetin downregulates Rac1 and NOX1 expression via generalized anti-inflammatory pathways rather than through direct binding [91]. In contrast, compounds like berberine have a closer interface with regulatory machinery by systematically depleting RhoA/ROCK activity to preserve blood–brain barrier (BBB) integrity [92]. For polyphenols such as epigallocatechin gallate (EGCG), direct biophysical evidence of binding remains limited, and their effects are largely secondary to upstream suppression of the NF-κB and MAPK pathways.

#### 3.1.3. Inhibition of Proinflammatory Cytokines:

Herbal compounds exert anti-inflammatory effects by downregulating the key cytokines, including TNF-α, IL-6, and IL-1β. By modulating signaling pathways such as NF-κB and JAK/STAT, these compounds control neuroinflammation and oxidative stress, ultimately reducing neuronal damage and protecting against neuronal dysfunction [93,94,95]. In addition, some extracts exhibit anti-inflammasome activity, reducing inflammasome activation and thereby mitigating inflammatory responses in the context of neurodegeneration [93].

#### 3.1.4. Regulation of Microglial Activation

Several phytochemicals help repress microglial overactivation and prevent neuronal overactivity, thereby avoiding excessive neuroinflammation and neuronal injury [96,97]. For example, curcumin has been reported to suppress microglial NF-κB activation and promote a shift toward a less proinflammatory phenotype [98], whereas EGCG and quercetin have been associated with reduced cytokine release and attenuated microglial activation in experimental models [99,100]. Berberine has likewise been linked to inhibition of NF-κB/NLRP3 signaling and reduced glial inflammatory responses [101,102].

#### 3.1.5. Antioxidant and Mitochondrion Protection

Some phytochemical-based herbal extracts can enhance mitochondrial bioenergetics and reduce ROS production, thereby benefiting neuronal integrity and function. Ginsenoside Re, a saponin derivative of *Panax ginseng*, a triterpenoid, induces mitochondrial activity via upregulation of adenosine triphosphate (ATP) production and reduction of ROS production in neuronal cells [103]. Similarly, sulforaphane, an isothiocyanate present in cruciferous vegetables, activates the nuclear factor erythroid 2–related factor 2 (Nrf2) pathway, leading to increased levels of antioxidant enzymes and protection against mitochondrial oxidative injury [104]. Such metabolites are representative of how phytochemicals regulate mitochondrial activity and oxidative stress to support the neuronal ecosystem.

## 4. Important Herbal Compounds and Neuroprotective Effects

### 4.1. Curcumin (Curcuma longa) as a Neurotrophic Agent

Curcumin, the major polyphenolic compound and a major constituent of Curcuma longa (turmeric), curtails inflammatory, oxidative, and neuroplastic pathways, potentially exerting neuroprotective activity. Its pleiotropic activity has made it a candidate for investigation as a treatment for neuroinflammatory and neurodegenerative diseases.

#### 4.1.1. Inhibitors of the RhoA/ROCK Pathway and the BBB

Curcumin has demonstrated effects on the RhoA/Rho-associated coiled-coil-containing protein kinase signaling pathway, a major regulator of cytoskeletal dynamics, BBB permeability, and endothelial cell function [105,106]. In experimental studies, curcumin-induced inhibition of this pathway decreased endothelial tight junction disruption and leukocyte infiltration into the CNS, thereby reducing neuroinflammatory dysregulation and preserving neuronal architecture [107].

#### 4.1.2. Block of NF-κB and MAPK Signaling Pathways

Among the key drivers of neuroinflammation are the NF-κB and MAPK cascades, each of which induces the expression of proinflammatory cytokines such as TNF-α and IL-1β [108,109]. Curcumin exerts anti-inflammatory effects by inhibiting the phosphorylation and nuclear translocation of NF-κB, as well as the activation of extracellular signal-regulated kinases (ERK), JNK, and p38 MAPK [110]. This inhibition results in limited microglial activation and cytokine expression in the neurodegenerative disease models [111,112].

#### 4.1.3. Promoting Neurogenesis and Synaptic Plasticity

Curcumin activates and enhances neurogenesis and synaptic plasticity by upregulating brain-derived neurotrophic factor (BDNF) expression. BDNF is necessary for neuron survival, development, and function. In rodent models of AD, curcumin increases hippocampal BDNF levels and improves learning and memory [113,114].

#### 4.1.4. Curcumin-Induced Reduction of Oxidative Stress

Curcumin is a powerful antioxidant that directly scavenges ROS and stimulates the expression of endogenous antioxidant enzymes, such as superoxide dismutase (SOD), catalase, and glutathione peroxidase. This redox modulation alleviates mitochondrial dysfunction and lipid peroxidation, thereby protecting neurons from oxidative injury [115].

#### 4.1.5. Interaction of Microglial Activation

CNS resident immune cells, microglia, play a dual role in neuroinflammation. Curcumin induces a shift in microglial phenotype from proinflammatory M1 to neuroprotective M2. This immunomodulatory phenomenon leads to decreased release of neurotoxic mediators and aids the resolution of chronic neuroinflammation [115,116].

**Figure 2:**
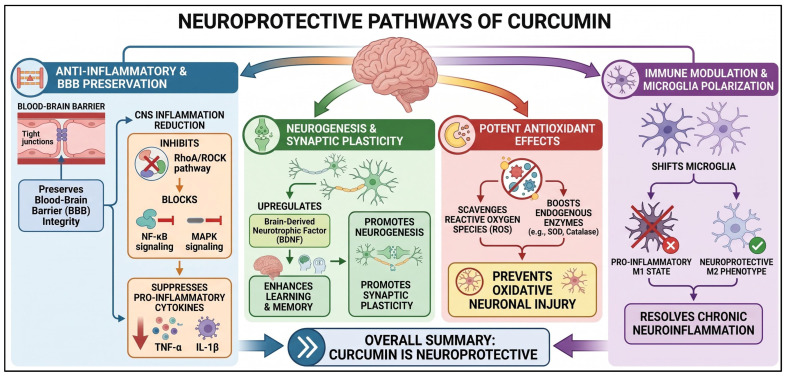
Multifaceted neuroprotective mechanisms and signaling pathways of curcumin. The schematic illustrates the therapeutic orchestration of curcumin across distinct cellular and molecular cascades to achieve overall neuroprotection in the CNS. Curcumin acts as a comprehensive neuroprotective agent against neurodegenerative pathologies through these concurrent anti-inflammatory, antioxidant, neuroplastic, and immunomodulatory pathways.

In summary (Figure 2), curcumin exerts neuroprotective effects by targeting key inflammatory, oxidative, and neuroplastic pathways. It preserves blood–brain barrier integrity and reduces CNS inflammation by inhibiting the RhoA/ROCK pathway and blocking NF-κB and MAPK signaling, thereby suppressing proinflammatory cytokines. Additionally, curcumin promotes neurogenesis and synaptic plasticity by upregulating brain-derived neurotrophic factor (BDNF), which enhances learning and memory. As a potent antioxidant, it scavenges reactive oxygen species and boosts endogenous enzymes to prevent oxidative neuronal injury. Finally, curcumin modulates immune responses by shifting microglia from a proinflammatory M1 state to a neuroprotective M2 phenotype, helping to resolve chronic neuroinflammation.

### 4.2. Resveratrol (Vitis vinifera) and Its Neuroprotection

Resveratrol is perhaps the most researched polyphenol for brain health, as it crosses the blood–brain barrier and addresses multiple avenues of degenerative cellular processes [117].

#### 4.2.1. Modulation of Rac1 and NADPH Oxidase (NOX)

Resveratrol’s mechanism of action through oxidative stress inhibition is beyond passive blockade; it involves active regulation of the NOX complex via a sophisticated molecular pathway that activates AMP-activated protein kinase (AMPK). This activation of AMPK acts as a negative regulator of Rac1 [86], a crucial cytosolic subunit critical for the formation of the active NOX complex, thereby preventing its membrane translocation and, consequently, its interaction with other subunits, including p67phox [118]. By inhibiting NOX enzyme assembly through this mechanism, resveratrol also induces a marked reduction in superoxide anion (O_2_∙^−^) production, the cell’s main oxidative byproduct [119]. Additionally, the simultaneous upregulation of endogenous antioxidant enzymes, such as manganese superoxide dismutase (SOD2), further enhances this reaction; SOD2 neutralizes reactive oxygen species and maintains cellular stability by removing them from the bloodstream.

#### 4.2.2. Cytokine Regulation (TNF-α and IL-6)

Resveratrol acts as an effective anti-inflammatory agent by disrupting signaling pathways that normally promote proinflammatory gene expression, primarily by suppressing the transcription factor NF-κB [120,121]. The degradation of its inhibitor, NF-κB inhibitor (IκB), hinders the transcriptional translocation of the p65 subunit. Resveratrol deacetylates the p65 subunit, allowing sirtuin 1 (SIRT1) to be activated, which plays an important role in suppressing proinflammatory cytokines by physically preventing p65 from binding to DNA [122] and by suppressing transcription of proinflammatory cytokines such as TNF-α and IL-6, thus alleviating the cytokine storm in the context of neurodegeneration.

#### 4.2.3. The Connection Between Mitochondrial Biogenesis and Neuronal Survival

With respect to mitochondrial biogenesis and neuronal survival, resveratrol is essential for strengthening neuronal resilience by inducing the formation of new, healthy mitochondria via the SIRT1/PGC-1α axis [123,124]. This pathway involves SIRT1-mediated deacetylation and activation of peroxisome proliferator-activated receptor γ coactivator 1-α (PGC-1α), followed by nuclear respiratory factor 1 (Nrf1), nuclear respiratory factor 2 (Nrf2), and mitochondrial transcription factor A (TFAM) [123]. The ultimate harbinger, TFAM, comes to mitochondria to initiate the replication of mtDNA to give rise to increased mitochondrial density that provides a steady supply of ATP and improved calcium buffering and is associated with a decrease in neuronal excitotoxicity and, by that, with protection from Caspase-3, which is the main promoter at the time of programmed cell death.

**Figure 3:**
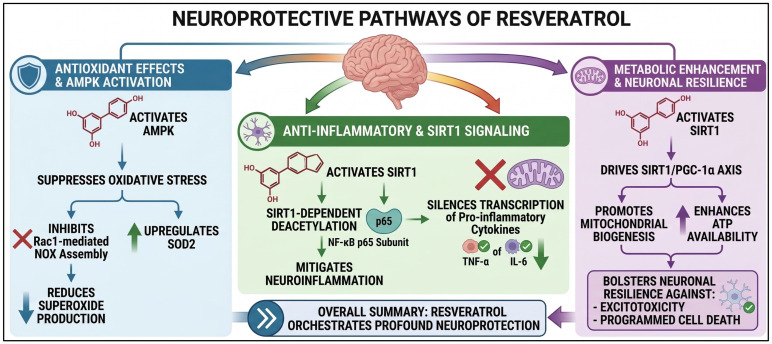
Multifaceted neuroprotective mechanisms and signaling pathways of resveratrol. The schematic illustrates the comprehensive therapeutic mechanisms of resveratrol within the CNS, orchestrating a protective response across parallel oxidative, inflammatory, and metabolic pathways. Resveratrol orchestrates a profound multi-target neuroprotective defense system by integrating antioxidant, anti-inflammatory, and metabolic-stabilizing cascades.

In summary (Figure 3), resveratrol exerts profound neuroprotection by orchestrating a multifaceted response across oxidative, inflammatory, and metabolic pathways. It actively suppresses oxidative stress by activating AMPK, which inhibits Rac1-mediated NOX assembly, thereby reducing superoxide production, while concurrently upregulating SOD2. Furthermore, resveratrol mitigates neuroinflammation via SIRT1-dependent deacetylation of the NF-κB p65 subunit, thereby silencing the transcription of proinflammatory cytokines such as TNF-α and IL-6. Finally, by activating the SIRT1/PGC-1α axis, resveratrol promotes mitochondrial biogenesis and increases ATP availability, thereby bolstering neuronal resilience against excitotoxicity and programmed cell death.

### 4.3. Ginsenosides (Panax ginseng) and Their Neuroprotective Capability

Ginsenosides, the primary bioactive constituents of *Panax ginseng*, including Rg1 and Rb1, exert neuroprotective effects by modulating Rho-family GTPases, specifically the RhoA/ROCK pathway, thereby facilitating cytoskeletal reorganization and axonal growth [125]. These compounds effectively suppress proinflammatory mediators, including cyclooxygenase-2 (COX-2) and inducible nitric oxide synthase (iNOS), in activated microglia while simultaneously strengthening BBB integrity to preserve the neurovascular unit and enhance functional recovery following injury [126].

#### 4.3.1. Inhibition of Pro-Inflammatory Mediators

Ginsenosides such as Rb1, Rg1, Rg3, and Rh2 effectively attenuate the production of proinflammatory cytokines, including TNF-α, IL-1β, and IL-6, by inhibiting the activation of the NF-κB and MAPK signaling pathways [127,128]. By downregulating these crucial signaling cascades, ginsenosides prevent excessive inflammation in neuronal tissues, which neurodegenerative processes typically exacerbate.

#### 4.3.2. Ginsenosides-Induced Modulation of Microglial Activation

As primary immune cells of the CNS, microglia are shifted from a proinflammatory M1 phenotype to an anti-inflammatory M2 phenotype by ginsenosides. This transition reduces neurotoxic factors and increases anti-inflammatory cytokine secretion, promoting the resolution of neuroinflammation [129].

#### 4.3.3. Regulation of Oxidative Stress

Ginsenosides demonstrate potent antioxidant properties by directly scavenging ROS and upregulating endogenous antioxidant enzymes, thereby shielding neurons from the oxidative damage that significantly contributes to neuroinflammatory conditions and supporting overall brain health [130].

#### 4.3.4. Increased Survival and Function of the Neurons

In addition to their contribution to alleviating inflammation and oxidative stress, these compounds promote neurogenesis and synaptic plasticity, thereby enhancing cognitive performance across a wide range of neurodegenerative models by modulating survival signaling and inhibiting apoptosis [130,131,132]. Some of these neuroprotective effects are mediated through precise modulation of long-term neuronal resilience and functionally viable cell signaling pathways.

**Figure 4:**
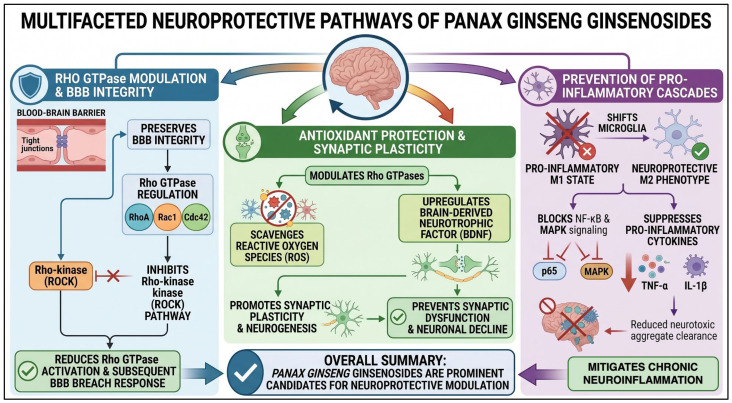
Multifaceted neuroprotective pathways of Panax ginseng ginsenosides. The schematic illustrates the complex therapeutic orchestration of *Panax ginseng* ginsenosides within the CNS. Ginsenosides mitigate neuroinflammation and prevent neuronal decline by cross-regulating structural barrier, oxidative, and proinflammatory signaling pathways. By concurrently modulating target Rho GTPase networks, enhancing antioxidant/synaptic defenses, and quenching inflammatory cascades, *Panax ginseng* ginsenosides emerge as highly promising candidates for neuroprotective interventions.

In summary (Figure 4), *Panax ginseng* ginsenosides show multifactorial therapeutic activity by modulating Rho GTPase activity, enhancing antioxidant protection, and preventing proinflammatory cascades, making them prominent candidates for neuroprotective modulation of neuroinflammation and subsequent neuronal decline.

### 4.4. Epigallocatechin Gallate (EGCG, Green Tea)

EGCG, a common catechin in green tea (*Camellia sinensis*), has attracted considerable interest due to its neuroprotective effects, including modulation of Rho family GTPases and reduction of neuroinflammation. Multiple mechanisms underpin its potential for treating various neurodegenerative diseases with numerous therapeutic effects.

#### 4.4.1. Inhibition of Pro-Inflammatory Signaling Pathways

EGCG has been shown to block the activation of key proinflammatory pathways, notably NF-κB and MAPKs [89]. By inhibiting these pathways, EGCG reduces the expression of proinflammatory cytokines, such as TNF-α and IL-6, in the brain [133].

#### 4.4.2. EGCG-Induced Modulation of Microglial Activation

Microglia, the CNS resident immune cells, are key players in neuroinflammation. EGCG drives a transition from proinflammatory (M1) toward anti-inflammatory (M2) expression, thereby modulating microglial activation [88]. By inhibiting these pathways, EGCG reduces the expression of proinflammatory cytokines such as TNF-α and IL-6 in the brain [134].

#### 4.4.3. EGCG-Induced Reduction of Oxidative Stress

Neurodegenerative disease has a considerable contribution of oxidative stress to neuronal injury. EGCG has strong antioxidant effects by removing ROS and upregulating endogenous antioxidant mechanisms [135]. Such an action protects the neurons against oxidative damage and benefits their health.

#### 4.4.4. Inhibition of Protein Aggregation

Protein misfolding and aggregation are among the most common pathological characteristics of diseases like AD and PD. EGCG also inhibits Aβ and α-synuclein accumulation, leading to toxic oligomer and fibril formation [136]. This inhibition can help preserve protein homeostasis and minimize its neurotoxicity.

#### 4.4.5. Modulation of Rho GTPase Activity

Although direct evidence of EGCG binding to Rho GTPases is limited, EGCG may influence cytoskeletal dynamics and cellular communication, making it a candidate for modulating protein function. EGCG may play important roles in stabilizing the cytoskeleton, augmenting blood–brain barrier integrity, and mitigating leukocyte infiltration through pathways upstream or downstream of Rho GTPases, all of which are necessary for regulating neuroinflammation [137].

**Figure 5:**
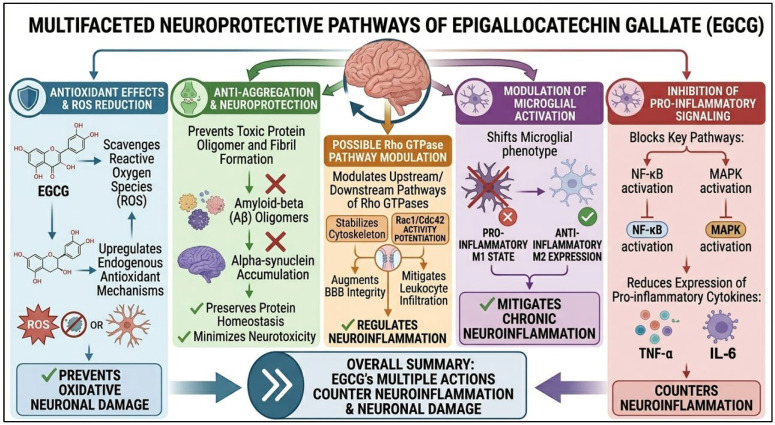
Multifaceted Neuroprotective Pathways of Epigallocatechin Gallate (EGCG). The schematic illustrates the complex therapeutic mechanisms of epigallocatechin gallate (EGCG) within the CNS. EGCG coordinates a multi-targeted defense across distinct signaling pathways to counter neuroinflammation, oxidative stress, and proteotoxicity. EGCG’s combined anti-inflammatory, antioxidant, anti-aggregation, and structural-stabilizing effects offer a holistic therapeutic strategy to address neuroinflammation and neuronal damage.

In summary (Figure 5), the neuroprotective effects of EGCG result from anti-inflammatory, antioxidant, and anti-aggregation effects, as well as possible modulation of pathways associated with Rho GTPases. The multiple actions of EGCG also make it an appealing therapeutic approach for countering neuroinflammation and neuronal damage.

### 4.5. Berberine (Berberis Species)

Berberine, a strong isoquinoline alkaloid and a product of the tree genus Berberis, possesses a neuroprotective effect by downregulating the expression of RhoA, which inhibits the degradation of tight junction protein within the blood–brain barrier via its ability to block the degradation of the tight junction protein. In addition, it attenuates neuroinflammation by inhibiting NF-κB signaling, suppressing the nucleotide-binding oligomerization domain leucine-rich repeat and pyrin domain-containing 3 (NLRP3) inflammasome, and downregulating neurotoxic cytokines such as IL-1β. The compound also suppresses microglial overactivation through modulation of Rho GTPase signaling, which reduces microglial overstimulation through modulation of Rho GTPase signaling (diminishing microglial overdrive). The combined effect of these two processes creates a neuroprotective environment that improves learning and memory performance in neurodegenerative models, ultimately providing long-lasting effects.

#### 4.5.1. Modulation of Rho GTPase Signaling and BBB Integrity

Berberine acts by downregulating RhoA activity and its downstream effector ROCK, thereby maintaining the structural integrity and functionality of the CNS. In the setting of neuroinflammation, this pathway typically enhances cytoskeletal instability, leading to BBB obstruction and leukocyte infiltration. In contrast, berberine-mediated downregulation of RhoA/ROCK decreases vascular permeability, thereby reducing BBB injury and lessening neuronal apoptosis [101].

#### 4.5.2. Inhibition of NF-κB and NLRP3 Inflammasome Activation

Berberine is a powerful anti-inflammatory agent that prevents NF-κB translocation and the activation of the NLRP3 inflammasome [138]. Its inhibition not only prevents pyroptosis [138] but also inhibits the primary drivers of neuroinflammation by reducing the release of pro-inflammatory cytokines (TNF-α, IL-1β, and IL-6). This broad inhibition of inflammatory signaling reduces excessive microglial activation, which is a critical driver of the pathogenesis of chronic neurodegenerative disease. Within that framework, berberine is a medication that could help reduce excessive microglial cell activation, a key factor in chronic neuroinflammation in neurodegenerative disorders, by downregulating these cells.

#### 4.5.3. Increased Synaptic Plasticity and Cognitive Function

Berberine may also affect cognitive resilience by modulating Rho GTPases, specifically by increasing Rac1 levels, thereby enhancing dendritic spine stability and synaptic plasticity. This cytoskeletal reorganization occurs in AD models, with remarkably large effects on learning and memory performance [139]. Furthermore, berberine enhances its neuroprotective potential through oxidative stress-scavenging actions. By directly scavenging ROS and activating mitochondrial function to sustain oxidative ATP generation, berberine protects against oxidative injury in AD and PD. Given berberine’s general ability to directly regulate Rho GTPase signaling by downregulating the NF-κB/NLRP3 axis and upregulating antioxidant defenses, it has established itself as a candidate drug for neurodegenerative diseases.

#### 4.5.4. Protection Against Oxidative Stress and Mitochondrial Dysfunction

Berberine exerts significant neuroprotective effects by modulating Rho-dependent oxidative stress responses that drive cellular oxidative stress and by facilitating antioxidant-associated activities in the CNS. Berberine exhibits strong antioxidant activity by directly scavenging reactive oxygen species, efficiently mitigates oxidative stress-induced neuronal injury, and protects cells from deterioration of cellular structure induced by neurodegenerative stressors [101]. Moreover, berberine enhances mitochondrial function by preserving ATP production, stabilizing mitochondrial membrane potential, and suppressing the signaling cascade that mediates neuronal apoptosis. Taken together, the synergistic effects of berberine attenuate oxidative injury and enhance mitochondrial bioenergetics, providing multifunctional protection against the oxidative damage associated with AD- and PD-related pathologies.

**Figure 6:**
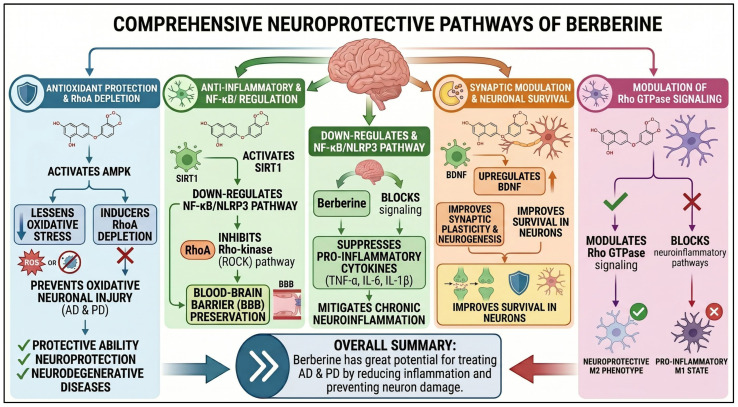
Neuroprotective pathways of berberine in neurodegenerative diseases. The schematic illustrates the multi-targeted therapeutic mechanism of berberine within the CNS, highlighting its capacity to counteract neuroinflammation, oxidative injury, and synaptic decline in diseases such as AD and PD. By depleting RhoA, downregulating the NF-kB/NLRP3 pathway, modulating synaptic function, and alleviating oxidative stress, berberine holds significant therapeutic potential for treating AD and PD by reducing neuroinflammation and preventing neuronal damage.

In summary (Figure 6), these effects enhance berberine’s protective capacity against oxidative injury in diseases such as AD and PD. Berberine has the potential to modulate Rho GTPase signaling, block neuroinflammatory pathways, and enhance neuronal survival. These effects make it a very likely option for treating neurodegenerative diseases. Berberine exerts profound neuroprotective effects by depleting RhoA, downregulating NF-κB/NLRP3, modulating synaptic function, and reducing oxidative stress.

### 4.6. Quercetin (A Flavonoid Found in Various Plants)

Quercetin exerts its neuroprotective effects by inhibiting the buildup of ROS via Rac1, thereby attenuating the effector of microglial activation and initiating a neuroinflammatory cascade. By regulating these Rho GTPase signaling cascades, it aims to stabilize neuronal cytoskeletons and improve synaptic function, particularly in AD. Furthermore, quercetin enhances cellular resilience by providing antioxidant protection and reducing oxidative stress, which drives neuronal degeneration in several neurodegenerative diseases.

#### 4.6.1. Inhibition of Rac1-Mediated ROS Production

Quercetin, one of the many flavonoids of natural origin, could exert remarkable protection in the brain by modulating Rho family GTPases, notably by downregulating Rac1, thereby reducing ROS production [140,141]. In the CNS, Rac1 signaling is the key driver of NADPH oxidase complex formation, a pathway implicated in oxidative stress and neuronal degeneration in AD and PD [142,143,144]. This downregulation of Rac1 activation and its downstream oxidative cascade explains quercetin’s ability to block mitochondrial dysfunction and concomitantly protect neurons from the cytotoxic effects of neuroinflammation and ischemic insult [140]. Furthermore, quercetin regulates Rho GTPases, maintains neuronal cytoskeleton stability, and increases synaptic plasticity, thereby enhancing cognitive resilience in experimental neurodegenerative models.

#### 4.6.2. Suppression of Microglial Activation and Neuroinflammation

Microglial cells are key immune cells of the CNS that often become activated and produce excessive amounts of proinflammatory cytokines in response to neuroinflammation [50]. This mechanism has two main components: the RhoA/ROCK pathway, an important molecular regulator of microglial activation and morphological transformation. Thus, quercetin inhibits RhoA/ROCK signaling, leading to reduced microglial activation and a transition to a mostly neuroprotective cellular state [145]. Quercetin modulates key inflammatory mediators, such as TNF-α, IL-1β, and IL-6, by inhibiting their pathways and can effectively suppress detrimental neuroinflammatory responses in the brain [146]. Additionally, in several disease models, quercetin can reduce glial scar formation and neuronal loss, thereby aiding tissue restoration and recovery [147,148].

#### 4.6.3. Increase in Synaptic Plasticity and Neuroprotection

Rho GTPases, particularly Rac1 and CDC42, play a pivotal role in modulating synaptic plasticity through dendritic spine assembly and actin cytoskeletal remodeling. As a consequence, disruption of these two distinct neurotransmission pathways is crucial to the pathophysiology of neurodegenerative diseases, characterized by gradual, progressive cognitive impairment. Quercetin activation of CDC42 and Rac1 has also been reported to stimulate synaptic remodeling, leading to the stabilization of dendritic spines [149]. Quercetin administration significantly influences memory and learning performance in AD under experimental conditions by modulating several key synaptic signaling pathways [150]. The same research shows that the neuroprotective effects of quercetin correlate with the durability of long-term potentiation, a primary cellular architecture for memory formation [151,152].

#### 4.6.4. Protecting the Brain Against the BBB

The structural integrity of the BBB is the most critical element of CNS homeostasis [53,153]. Previous studies demonstrate that quercetin, by blocking RhoA/ROCK-mediated endothelial permeability, does not significantly compromise BBB integrity in MS and ischemic stroke [152,154]. Moreover, quercetin increases tight junction proteins, including occludin and claudin-5, thereby strengthening the barrier and protecting the BBB against neuroinflammation [155,156].

#### 4.6.5. Autophagy Regulation and Survival of Neurons

Dysfunction of autophagy is one of the most powerful mechanisms in neurodegenerative disease, since Rho GTPases are important molecular players to modulate the balance between autophagic clearance and apoptotic signaling [157,158]. In addition, robust evidence demonstrates that quercetin activates autophagic signaling pathways, such as the SIRT1/AMPK/mTOR axis; activation of this pathway increases the degradation kinetics of toxic protein aggregates, including amyloid-beta and alpha-synuclein, in AD and PD models [159,160]. Moreover, quercetin suppresses apoptosis through the PI3K/Akt and Rho GTPase signaling pathways and targets pro-apoptotic signaling pathways, thereby increasing brain longevity and structural stability by protecting against neuronal apoptosis [161,162].

**Figure 7:**
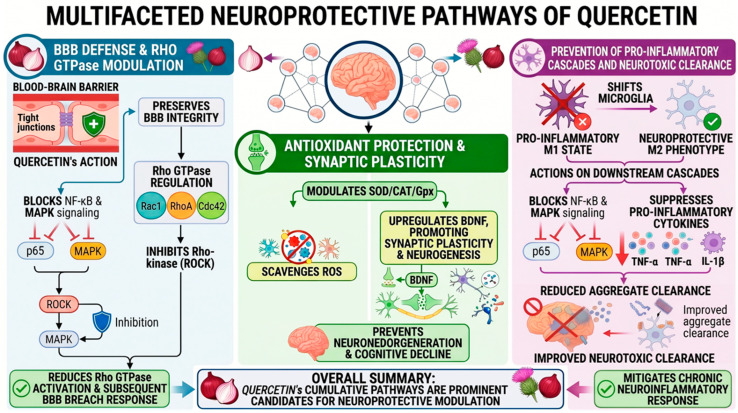
Comprehensive neuroprotective pathways of quercetin in CNS disorders. The schematic outlines the multi-targeted therapeutic mechanisms of quercetin within the CNS, highlighting its capacity to modulate Rho GTPase signaling, reduce neuroinflammation, and enhance neuronal survival in disorders such as AD, PD, and MS. Quercetin shows great clinical promise for the treatment of AD, PD, and MS via its ability to cross-regulate Rho GTPase signaling, augment baseline neuroprotection, and quell chronic neuroinflammatory cascades.

In summary (Figure 7), the potential of quercetin to modulate Rho GTPase signaling makes it an attractive drug-class target for neuroinflammatory and neurodegenerative disorders. Quercetin exhibits neuroprotective effects through Rac1 inhibition, suppression of microglial activation, enhancement of synaptic plasticity, BBB protection, and modulation of autophagy. Additional clinical studies are needed to assess its therapeutic potential for brain diseases, such as AD, PD, and MS.

### 4.7. Other Herbal Compounds with Neuroprotective Potential

**Figure 8:**
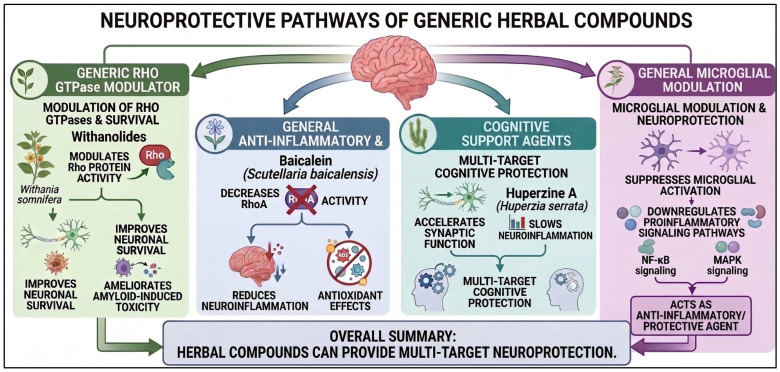
Multi-targeted neuroprotective pathways of diverse herbal compounds. This schematic illustrates how distinct plant-derived bioactives safeguard the CNS. Specifically, withanolides and baicalein modulate Rho GTPase signaling (Rho/RhoA) to improve neuronal survival and suppress neuroinflammation. Concurrently, huperzine A enhances synaptic function to provide cognitive protection, while andrographolide downregulates NF-κB and MAPK pathways to inhibit microglial activation. Together, these compounds offer significant therapeutic potential by counteracting chronic neuroinflammation and promoting overall neuroprotection.

In addition, various herbal compounds demonstrate significant neuroprotective potential by modulating Rho GTPases and neuroinflammatory pathways (Figure 8). Withanolides isolated from *Withania somnifera* can modulate Rho GTPase activity [163], thereby improving neuronal survival and ameliorating amyloid-induced toxicity and subsequent neuronal injury. Likewise, baicalein, present in *Scutellaria baicalensis*, has clear anti-inflammatory and antioxidant effects [164]; this may be mediated by a specific decrease in RhoA activity, thereby reducing neuroinflammation [165]. In addition, *Huperzia serrata*-derived huperzine A, another compound, has been shown to accelerate synaptic function, slow neuroinflammation, and provide multi-target cognitive protection [166,167]. Lastly, andrographolide, a bioactive constituent indicator of Andrographis paniculata, suppresses microglial activation and provides general neuroprotection by acting as an effective anti-inflammatory/protective agent through downregulation of proinflammatory signaling pathways [168,169,170].

## 5. Future Perspectives

As a conclusion to the molecular interactions discussed in the preceding sections, Table 1 provides a comprehensive synthesis of the phytochemicals, their primary Rho GTPase targets, and their evidence status, highlighting the structural diversity that must now be reconciled with systemic pharmacokinetics and translational challenges. Innovative therapeutics should prioritize cellular and contextual specificity by elucidating regulatory mechanisms that selectively modulate Rho GTPases in active microglia while preserving the critical homeostatic roles of neighboring neurons and astrocytes. Despite promising preclinical profiles, the clinical translation of compounds such as curcumin, resveratrol, and EGCG is fundamentally hindered by poor pharmacokinetic properties, including exceptionally low aqueous solubility, rapid phase II metabolism in the intestine, and low systemic bioavailability. Furthermore, under physiological conditions, the highly restrictive blood–brain barrier (BBB) actively excludes these hydrophobic clusters from achieving therapeutic thresholds in the central nervous system (CNS). Advanced drug delivery systems—such as solid lipid nanoparticles (SLNs), polymer-curcumin conjugates, or exosome-mediated vehicles—are essential for shielding these molecules from premature degradation and exploiting active endothelial transcytosis, bridging the gap between benchtop efficacy and human clinical reality. By delivering therapeutic concentrations of bioactive molecules (e.g., quercetin and andrographolide) to the CNS, these systems significantly enhance the efficacy of current therapies. Moreover, a network pharmacology and systems biology perspective will facilitate a more holistic map of additive and antagonistic interactions among phytochemicals and the complex Rho GTPase signaling network. These approaches may eventually yield healthier therapies for more complex disorders. It will now be essential to clinically establish the safety and effectiveness of these herbal agents through long-term, comprehensive clinical trials and to develop this approach to early detection as a solution for the early phase of neurodegenerative disease in human populations.

## 6. Conclusions

In summary, this review highlights Rho family proteins as major regulators of the neuroinflammatory system with critical roles in neurodegenerative diseases. Dysregulation of these GTPases—overactivity of RhoA/ROCK signaling and suppression of Rac1/CDC42-mediated plasticity—is a main driver of microglial plastic hyperactivation, blood–brain barrier damage, and synaptic destruction. Herbal products such as quercetin, withanolides, baicalein, huperzine A, and andrographolide have emerged as highly promising in restoring this molecular homeostasis. These findings enable the natural agents to dampen the release of proinflammatory cytokines (TNF-α, IL-1β, IL-6), amplify autophagic clearance of toxic aggregates, and stabilize the dendritic cytoskeleton by suppressing the RhoA/ROCK axis and potentiating Rac1/CDC42 activity. Collectively, available evidence supports Rho GTPases as promising therapeutic targets rather than definitive drug targets. Current findings remain predominantly derived from in vitro and animal studies, whereas direct clinical evidence demonstrating modulation of Rho signaling by phytochemicals remains limited. Furthermore, the biological effects of Rho GTPase modulation appear highly context-dependent, varying according to disease stage, cell type, and microenvironment. Consequently, future investigations should integrate mechanistic validation, pharmacokinetic optimization, and clinical translation before phytochemical-based Rho GTPase modulation can be considered a viable therapeutic strategy.

## Figures and Tables

**Table 1:** Comprehensive summary of phytochemical modulators targeting Rho GTPase signaling pathways, downstream networks, and their current level of experimental evidence.

Phythonchemicals	Primary Rho GTPase Target(s)	Key Downstream/Intersecting Pathways	Mechanism Status	Supporting Evidence Level
Curcumin (*Curcuma longa*)	RhoA (Inhibition)	ROCK; NF-κB; MAPK (ERK; JNK; p38); BDNF	Indirect/Upstream & Downstream Cascade	Experimental/Rodent Models
Resveratrol (*Vitis vinifera*)	Rac1 (Negative Regulation)	AMPK (Upstream activator); NOX assembly; SIRT1/PGC-1α	Indirect via AMPK activation	Experimental/Cellular Models
Ginsenosides (*Panax ginseng*)	RhoA/ROCK (Modulation)	COX-2; iNOS; NF-κB; MAPK	Upstream Signaling Network	Experimental/Neurodegenerative Models
EGCG (Green Tea)	Upstream/Downstream of Rho family	NF-κB; MAPKs; Aβ & α-synuclein aggregation	Indirect (Direct binding evidence is limited)	Experimental/Microglial Lineages
Berberine (*Berberis* spp.)	RhoA (Downregulation); Rac1 (Upregulation)	ROCK; NF-κB; NLRP3 Inflammasome	Structural Alignment & Pathway Depletion	Experimental/AD & PD Models
Quercetin (Flavonoid)	Rac1 (Downregulation); RhoA/ROCK (Inhibition); Cdc42 (Activation)	NADPH Oxidase (NOX); SIRT1/AMPK/mTOR; PI3K/Akt	Multi-targeted Enzymatic Interference	Experimental/Ischemic & Neurodegenerative Models
Baicalein (*Scutellaria baicalensis*)	RhoA (Decrease)	Macrophage efferocytosis & polarization	Pathway-Specific Reduction	Experimental/Cellular Models
Withanolides (*Withania somnifera*)	Rho family proteins (Modulation)	Amyloid-induced toxicity attenuation	General Protein Activity Modulation	Experimental Models

## Data Availability

No new data were created or analyzed in this study. Data sharing is not applicable to this article.

## Abbreviations

The following abbreviations are used in this manuscript:

AD	Alzheimer’s disease
PD	Parkinson’s disease
MS	multiple sclerosis
TBI	traumatic brain injury
RhoA	Rho family protein (Ras homolog family member A
Rac1	Ras-related C3 botulinum toxin substrate 1
CDC42	cell division control protein 42 homolog
BBB	blood–brain barrier
Aβ	amyloid-beta
CNS	central nervous system
MHC II	major histocompatibility complex class II
TNF-α	tumor necrosis factor-alpha
IL-6	interleukin-6
IL-1β	interleukin-1 beta
NF-κB	nuclear factor kappa B
JNK	c-Jun N-terminal kinases
MAPK	mitogen-activated protein kinase
ROCK	Rho-associated, coiled-coil-containing protein kinase
JAK	Janus kinase
STAT3	signal transducer and activator of transcription 3
ROS	reactive oxygen species
NADPH	nicotinamide adenine dinucleotide phosphate
ATP	adenosine triphosphate
Nrf1/Nrf2	nuclear factor erythroid 2–related factor 1/factor 2
ERK	extracellular regulated protein kinases
SOD	superoxide dismutase
NOX	NADPH oxidase
AMPK	AMP-activated protein kinase
SOD2	Manganese superoxide dismutase
IκB	inhibitor of NF-κB
SIRT1	sirtuin 1
TFAM	mitochondrial transcription factor A
COX-2	cyclooxygenase-2
iNOS	inducible nitric oxide synthase
EGCG	epigallocatechin gallate
NLRP3	nucleotide-binding oligomerization domain leucine-rich repeat and pyrin domain containing 3
